# Effect of Etching Temperature and Time on In Situ HF-Generating (NH_4_F) Synthesis of Ti_3_C_2_T_x_ MXene

**DOI:** 10.3390/ma19194172

**Published:** 2026-09-29

**Authors:** Dias Muldash, Mukhtar Alipuly, Aidyn Kazhmuratov, Shynggyskhan Sultakhan, Seitkhan Azat

**Affiliations:** Laboratory of Engineering Profile, Satbayev University, 22 Satbayev Street, Almaty 050013, Kazakhstan; muldashevdias@gmail.com (D.M.); mukhtaralipuly@gmail.com (M.A.); aidynkazhmuratov@gmail.com (A.K.)

**Keywords:** MXene synthesis, Ti_3_C_2_T_x_, in situ HF etching, ammonium fluoride (NH_4_F), microwave-assisted synthesis, phase transformation

## Abstract

**Highlights:**

**Main Findings**

- SEM shows favourable temperature and time at 80 °C, 30 min for NH_4_F/HCl etching.
- XRD composite index places the MAX-to-MXene crossover between 60 and 80 °C.
- EDS shows that the fluorine content and Al removal decouple with etching time.

**Abstract:**

MXenes are a family of two-dimensional transition-metal carbides and nitrides, produced by selectively etching the “A” layer from a layered MAX-phase ceramic precursor. Ti_3_C_2_T_x_ MXene was synthesised from Ti_3_AlC_2_ via microwave-assisted, in situ HF-generating etching using NH_4_F/HCl as a safer alternative to concentrated HF. The etching temperature was first varied (40–100 °C, 30 min), and then the duration (30–90 min) was varied at the most favourable temperature. Samples were characterised by SEM, EDS, XRD, and Raman spectroscopy. SEM showed a non-monotonic relationship between temperature and morphology, with the most uniform accordion-like layering at 80 °C before fragmentation and edge erosion at 100 °C; duration showed a similar pattern, with 30 min at 80 °C being most uniform and heterogeneity increasing at 60 and 90 min. XRD, using a composite residual-MAX-phase index, placed the MAX-to-MXene crossover between 60 and 80 °C, consistent with SEM. EDS showed fluorine content and aluminium removal decoupling with etching time, with fluorine declining sharply beyond 30 min without a corresponding recovery in aluminium removal. Raman confirmed the characteristic Ti_3_C_2_T_x_ fingerprint at every condition. Thus, 80 °C/30 min was identified as the most favourable processing window; however, given the coarseness of the parameter grid, the true optimum may lie within intermediate, untested conditions.

## 1. Introduction

### 1.1. Overview of 2D Materials and MXenes

Since the discovery of graphene, two-dimensional (2D) materials have become a hot topic in the research field of material science due to their unique properties [1,2]. This interest arose from their reduced dimensionality, including their high surface-area-to-volume ratios and flexible tunability of the surface chemistry [3]. Among the recently developed 2D materials are MXenes, which were first successfully synthesised in 2011 by Naguib et al. [2]. These are carbides, nitrides or carbonitrides that are exfoliated from the precursor known as the MAX-phase. They are ternary layered solids with the general formula of M_n+1_AX_n_ [4], where M is an early transition metal, A is a group 13 or 14 element, and X is carbon or nitrogen. Ti_3_C_2_T_x_ MXene is the most extensively studied type [2,5] because it was the first to be reported and because of the general accessibility of its precursor. Since then, MXenes have demonstrated applicability [6] in EMI shielding [7], energy storage [8], water treatment [9], and optoelectronic and photodetection devices [10,11]. This shows a broader trend across nanostructured transition-metal-based composites more generally toward high-performance electrochemical energy storage devices, as seen in unrelated systems such as CoO_x_/C_3_N_4_-derived supercapacitors [12].

### 1.2. MAX Phase to MXene Synthesis Mechanism

MAX phases cannot be mechanically exfoliated into their two-dimensional counterparts because the M-A metallic bond is significantly stronger than the interlayer forces holding the mechanically exfoliable materials together, such as the van der Waals forces between graphene sheets [13]. The M-A bond is still weaker than the M-X bond, which is what allows selective etching to remove the A layer while leaving the M-X framework intact. This process was the first general etching strategy that extended to other types of MXenes (such as Ti_2_C, Ta_4_C_3_, etc.) [14]. Etching leaves the exposed transition-metal surface terminated by functional groups: -O, -OH, and -F. They are collectively denoted T_x_ in the M_n+1_X_n_T_x_ notation. Terminations directly arise from the chemistry of the etchant and explicitly influence the resulting material’s properties [15], such as hydrophilicity, electrical conductivity, and electrochemical behaviour—an interface-dependent tunability conceptually similar to strategies used to engineer charge transport in unrelated nanostructured conductive systems, such as conducting-polymer bioelectrodes [16]. The product at the end of synthesis retains a stacked, accordion-like multilayer morphology, which can then be further delaminated into a single or few-layer nanosheet through intercalation and sonication [17,18].

### 1.3. Limitations of HF-Based Etching

In their original synthesis route [2], to extract the Al layer, Naguib et al. treated the MAX phase with concentrated hydrofluoric acid, exposing the Ti atoms to functionalisation by F atoms or hydroxyl groups upon exposure to water. Although this method is proven effective, it relies on the direct handling of concentrated HF, which can jeopardise people and environmental safety: HF is highly corrosive, causes deep tissue damage and systemic toxicity through dermal contact even at low concentrations [19], and requires stringent engineering controls and PPE [20]. Beyond safety, the aggressive nature of concentrated HF etching can generate a relatively high density of surface defects and less controllable etching kinetics, factors later shown to affect the oxidative stability of the resulting MXene [15,21].

These hazards motivated the development of etching strategies that utilise in situ methods, the most notable of them is the LiF + HCl system that was introduced by Ghidiu et al. [22]. Fluoride salts and hydrochloric acid are combined to gradually synthesise HF within the reaction mixture, instead of being introduced as is. This strategy was extended to make use of other fluoride-containing salts such as NaF and NH_4_F [23,24,25] and become a common route for achieving selective etching under comparatively milder and safer conditions. Still, the LiF + HCl route, while safer than direct HF use, requires long etching durations, often on the order of tens of hours [26,27], highlighting a need for etchant systems and process conditions that would balance the safety and efficiency of the synthesis process.

### 1.4. NH_4_F as an Alternative Etchant

Ammonium fluoride (NH_4_F) has emerged as one such alternative fluoride source for in situ HF generation. When combined with HCl, NH_4_F dissociates to release fluoride ions [23,28]. Then, they react with protons in solutions and gradually generate HF in a more controlled manner, allowing the avoidance of concentrated HF handling and transporting. Related studies have applied NH_4_F/HCl mixtures under hydrothermal [26] and microwave-assisted conditions [27] to etch Ti_3_AlC_2_, generally reporting the impact of morphology, composition, and physicochemical properties resulting from this in situ HF generation route comparable to, or more favourable than, conventional etching. Systems consisting of NH_4_F have also extended to more complex double-transition-metal MXene compositions [24], showing the versatility of the alternative etchant system across MAX phase chemistry. The NH_4_F method should not be interpreted as a pure “HF-free” method; rather, it is generated under acidic conditions, so appropriate safety precautions equivalent to those that are used for direct HF handling remain necessary [19,20].

### 1.5. Research Gap and Objectives

Despite the growth of the literature on the NH_4_F-based etching of Ti_3_AlC_2_, most protocols specify either a single fixed set of etching conditions [24,26,27] or multiple systems as a whole.

We are addressing the gap by investigating the combined effects of temperature and duration on the synthesis of Ti_3_C_2_T_x_ MXene from Ti_3_AlC_2_ via NH_4_F/HCl in situ HF generation while using the effectiveness of microwave-assisted etching [27]. By mapping how these two parameters jointly influence etching outcomes, this work aims to identify practical processing windows for NH_4_F-based, microwave-assisted MXene synthesis to contribute toward safer and time-efficient protocols for Ti_3_C_2_T_X_ production. Reproducibility remains a valid concern in MXene synthesis more broadly: recent work has shown that etching outcomes can depend on factors as subtle as surface passivation layers on air-stored MAX-phase precursor grains, with some widely cited synthesis routes proving difficult to reproduce independently [29]. While the individual effect of etching time has been investigated for NH_4_F/HCl-based microwave-assisted routes [27], and combined temperature–time optimisation has been reported for HF-based [30] and NH_4_HF_2_-based [31] systems, to our knowledge, no prior study has systematically varied both the etching temperature and duration within a microwave-assisted NH_4_F/HCl system specifically.

## 2. Materials and Methods

### 2.1. Precursor Materials and Reagents

The precursor material used for this experiment was Titanium aluminium carbide (Ti_3_AlC_2_) MAX-phase powder, manufactured by Y-Carbon LLC (Kyiv, Ukraine). Ammonium fluoride (NH_4_F) was produced by Meryer Chemical Technology Co., Ltd. (Shanghai, China)., and concentrated hydrochloric acid (HCl, ~37%, 12 M) was manufactured by Sigma-Aldrich (Darmstadt, Germany). For solution preparation and post-reaction washing, deionised (DI) water was used. Reagent handling and reaction steps involving acids were conducted in a fume hood with appropriate personal protective equipment, given that NH_4_F is capable of releasing HF under acidic conditions.

### 2.2. Ti_3_AlC_2_ Etching Procedure

By diluting concentrated HCl in DI water, 6 M HCl solution was prepared. After thorough mixing, the solution was allowed to equilibrate to room temperature prior to use (25 min). To prepare the etching mixture, 10 mL of the freshly prepared 6 M HCl was transferred into a microwave reactor vessel, manufactured by CEM (Matthews, NC, USA), and then, 1 g of NH_4_F was added to the vessel. The mixture was stirred until confirmation of complete dissolution of NH_4_F was achieved, which took 10 min from the moment of addition, as incomplete dissolution was found to result in non-uniform etching. After that, 0.5 g of Ti_3_AlC_2_ powder was introduced into the solution. The rate of the MAX-phase powder introduction was controlled at 0.1 g/min to ensure uniform contact between the MAX phase and the etchant and to avoid poorly reacting “lumps” of powder.

Following brief mixing, the mixture was then stirred pre-etching on a magnetic stirrer (YBOTECH, Hefei, China), which was conducted at 200 rpm for 15 min at room temperature, with the reactor cap left slightly ajar to permit pressure equalisation. This step was performed in an attempt to promote surface oxidation and activation of the MAX phase prior to microwave-assisted etching.

After sealing the vessels, they were transferred to a microwave reactor for the etching reaction, the specific parameters of which are detailed in Section 2.3. In all cases, the reactor was allowed to cool to ambient temperature, and atmospheric pressure was equilibrated before opening.

### 2.3. Experimental Design: Temperature and Time Matrix

To evaluate the influence of etching temperature and etching duration on Ti_3_C_2_T_x_ formation, two independent experimental series were conducted using a CEM MARS-6 Microwave Digestion System (Matthews, NC, USA) operated at 300 W with medium (dynamic) stirring setting under standard control mode. The temperature and time were varied sequentially (Table 1).

### 2.4. Washing and Drying Protocol

Following microwave-assisted etching and cooling to room temperature, the reactor vessel was opened inside a fume hood due to the presence of residual HCl and HF-generating species in the product mixture. The product suspension was transferred to 50 mL centrifuge tubes, with the reactor vessel rinsed with a small volume of DI water to recover residual material. The suspension was subjected to repeated centrifugation for 5 min per cycle, with the rotational speed increased incrementally by 200 rpm after each cycle (beginning at 2500 rpm), followed by decanting of the supernatant. Between cycles, the precipitate was gently redispersed by shaking. The pH of the supernatant was monitored after each wash cycle using pH paper, and the wash–centrifuge–decant process was repeated (typically 5–10 cycles) until a neutral pH of 6–7 was achieved, as residual acidity was expected to compromise MXene stability and surface chemistry [32]. In the final wash step, excess water was removed, leaving approximately 20 mL of suspension, which was gently redispersed and stored under refrigeration prior to freeze-drying. Samples were freeze-dried at −52 °C under a chamber pressure of 0.1 mbar for approximately one week to yield the dry Ti_3_C_2_T_x_ powder [33] used for subsequent characterisation. To minimise the influence of any single synthesis run, material from four independent batches per condition was pooled before characterisation.

### 2.5. Characterisation Technique

The morphology of the etched samples was examined using Quattro S environmental scanning electron microscopy (Thermo Fisher Scientific Waltham, MA, USA) to evaluate the extent of Al layer removal and the development of the characteristic accordion-like layered structure associated with successful MXene formation. Samples were imaged at an acceleration voltage of 20.00 kV and working distances from 10.62 mm to 9.57 mm, with representative micrographs collected across the sample surface to assess morphological uniformity as a function of etching temperature and time. The interlayer spacing was quantified from every clearly resolved interlamellar gap within one representative micrograph per condition (*n* = 12–14 measurements per condition); additional independent fields of view were not collected for this measurement due to instrument-time constraints, and this single-field-per-condition sampling is treated as a limitation of the reported spacing statistics (Section 3.1.2).

X-ray diffraction of the pristine Ti_3_AlC_2_ precursor was collected from the same commercial batch (Y-Carbon LLC) (Kyiv, Ukraine) used throughout the etching experiments, providing a direct experimental baseline for structural comparison in addition to the literature reference values. X-ray diffraction was used to track the structural evolution of the etched samples relative to the Ti_3_AlC_2_ precursor, in particular the attenuation of the characteristic (104) MAX-phase reflection and the shift and broadening of the (002) basal reflection as indicators of interlayer expansion and Al layer removal. Patterns were collected over a 2θ range of 5–80° using a Cu Kα radiation source (λ = 1.5406 Å) at a step size of 0.02°. The interlayer spacings were calculated from the position of the (002) basal reflection using Bragg’s law, nλ = 2d sinθ (*n* = 1; Cu Kα, λ = 1.5406 Å), and the c-lattice parameter was taken as c = 2d(002) for the hexagonal Ti_3_C_2_T_x_ structure. Prior to plotting, each pattern was normalised to its own mean background intensity over a flat, peak-free region (65–70° 2θ), consistent with the intensity-to-background convention used for the composite residual-MAX index. A composite residual-MAX index was calculated as the mean intensity-to-background ratio (I/background) across five non-basal reflections diagnostic of the MAX phase: (101), (104), (105), (107), and (109). Basal reflections (002) and (004) were excluded from this composite, as they are present in both the MAX and MXene phases and are therefore not diagnostic of conversion; their evolution was instead tracked separately via the (002) sharp/broad ratio. Raman spectroscopy was used to assess the structural evolution and surface-termination changes in the etched samples as a function of etching temperature. Spectra were collected on a Bruker Bravo spectrometer (Billerica, MA, USA) (Duo Laser™ excitation, 785 and 852 nm), which has a manufacturer-specified spectral range of 300–3200 cm^−1^, with the diagnostic Ti_3_C_2_T_x_ vibrational modes (E_g_ and A_1g_, below 800 cm^−1^) monitored to evaluate etching completeness and the carbon bonding environment across the experimental matrix [34].

## 3. Results

### 3.1. Morphological Evolution via SEM Analysis

SEM was used to evaluate the Al layer removal and the development of the accordion-like layered morphology characteristic of Ti_3_C_2_T_x_ across both the temperature series and the time series.

#### 3.1.1. Effect of Etching Temperature

**Figure 1:**
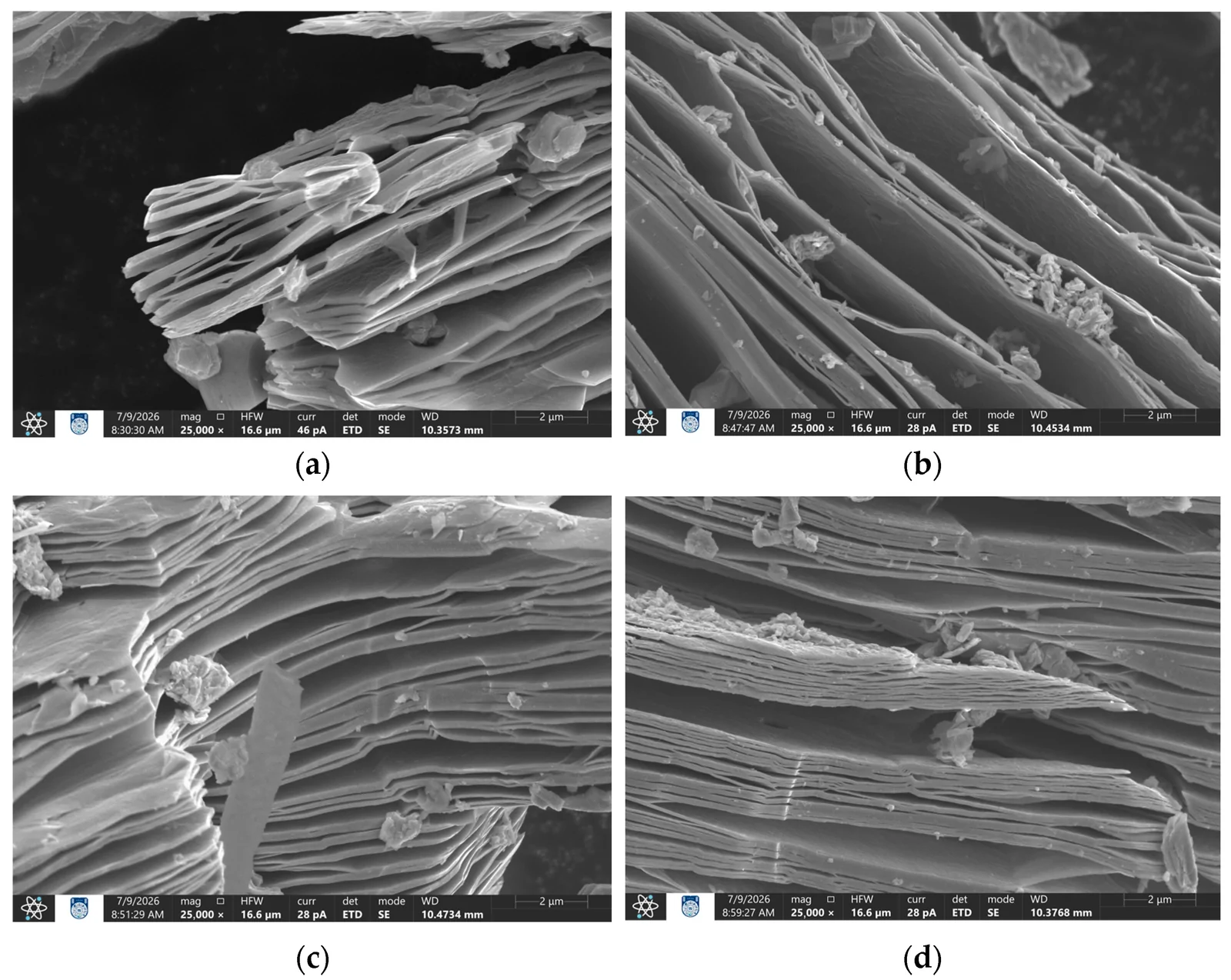
SEM micrographs of the etching temperature series, 25,000× magnification. (**a**) 40 °C. (**b**) 60 °C. (**c**) 80 °C. (**d**) 100 °C. Additional lower-magnification SEM survey images for each condition are provided in the Supplementary Materials (Figure S1).

At 40 °C, SEM images show the onset of accordion-like morphology, with well-resolved lamellae and interlayer gaps (Figure 1a).

At 60 °C, larger, more developed lamellar stacks are observed (Figure 1b), though dense, blocky, unlayered particles continue to coexist with well-etched regions.

For the results at 80 °C, the majority of particles exhibit visible lamellar structure (Figure 1c), representing a marked improvement in etching uniformity. The lamellae are thin, closely and uniformly spaced, and well-ordered, with minimal debris and no evidence of structural collapse or fragmentation.

At 100 °C, this trend reverses: imaging shows a field dominated by smaller, fragmented particles (Figure 1d). Sheet edges appear rougher and more frayed, with increased surface debris.

Across the 40–100 °C series, the etching outcome follows a clear non-monotonic trend: morphological quality improves progressively from 40 to 80 °C and then deteriorates at 100 °C due to particle fragmentation and edge erosion. On this basis, 80 °C was selected as the fixed temperature for the etching duration series.

#### 3.1.2. Effect of Etching Time (Experiment 2)

The 30 min time point corresponds to the 80 °C condition, which produced extensive, uniform accordion-like morphology (Figure 1c).

**Figure 2:**
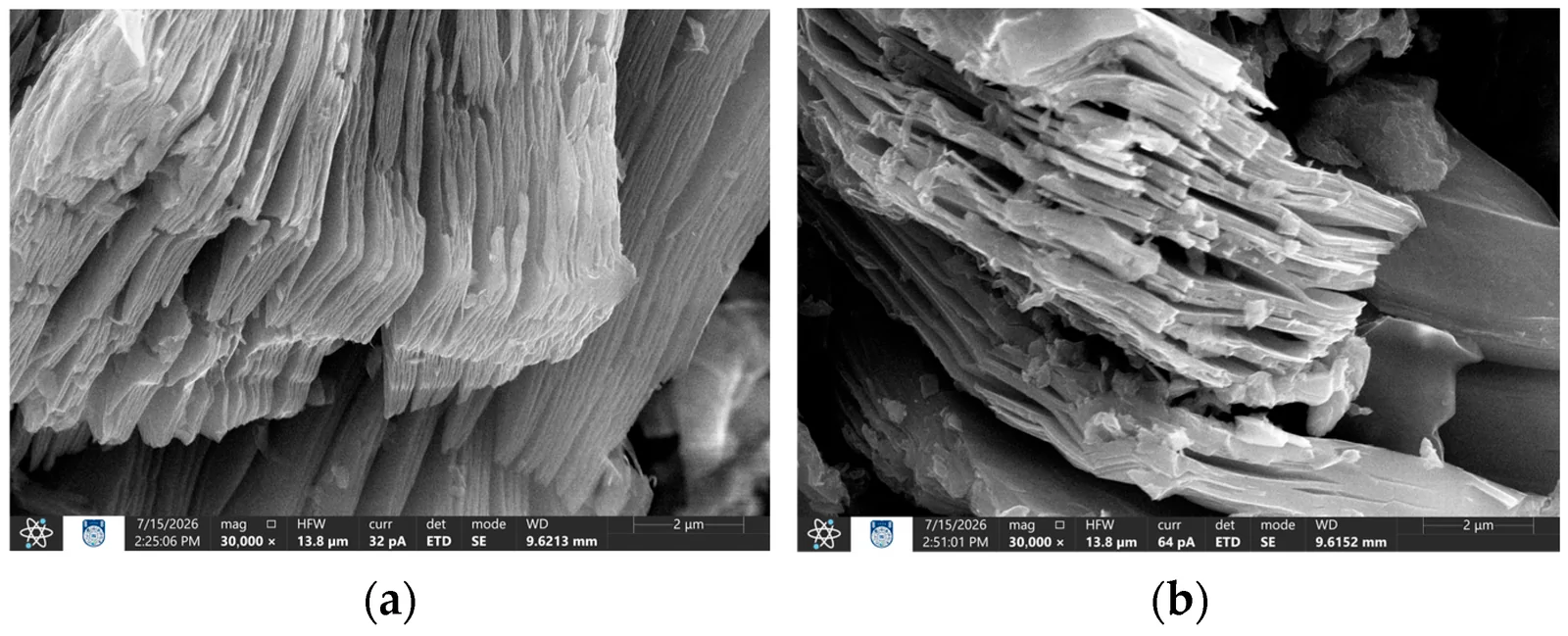
SEM micrographs of the etching duration series at 80 °C. (**a**) 60 min. (**b**) 90 min. Additional lower-magnification SEM survey images for each condition are provided in the Supplementary Materials (Figure S1).

At 60 min, a larger proportion of dense, blocky, poorly layered particles is evident. At higher magnification, some regions show sharply resolved lamellae, while others show wavy surfaces with less-defined boundaries (Figure 2a), indicating increased heterogeneity.

At 90 min, this heterogeneity intensifies: low-magnification imaging is dominated by dense, unlayered particles, with well-developed accordion structures confined to isolated regions (Figure 2b). Where present, lamellae are reasonably well-resolved but show rougher edges, resembling the edge irregularities seen in the over-etched 100 °C sample.

**Figure 3:**
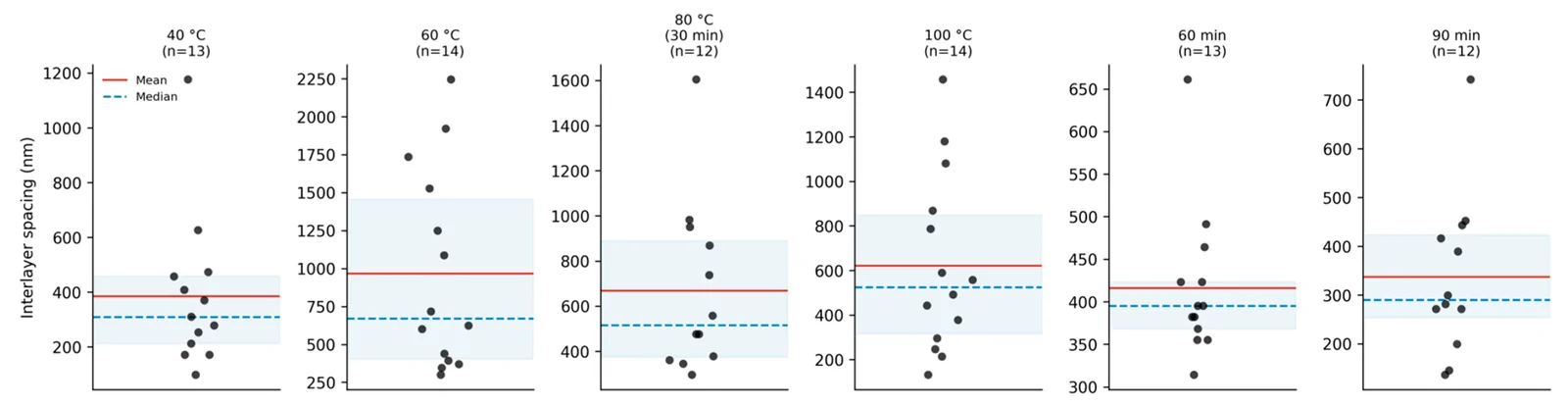
Individual SEM-derived interlayer-spacing measurements per condition, from every clearly resolved interlamellar gap in one representative micrograph per condition (*n* = 12–14; see Section 2.5). Blue shaded band indicates the interquartile range (IQR).

As described in Section 2.5, each condition’s measurements were drawn from every clearly resolved interlamellar gap in one representative micrograph; given the right-skewed distribution observed in every condition, the median and interquartile range (IQR) are reported, with the mean ± SD retained for comparison with the convention used elsewhere in the MXene literature. Every condition shows a right-skewed distribution of interlayer spacings, with the mean consistently exceeding the median (Table 2) because a small number of large, well-separated gaps sit well above a denser cluster of smaller values. This pattern is most pronounced at 60 °C (mean 967 nm vs. median 670 nm), where a subset of well-separated lamellae coexists with the dense layered particles noted qualitatively above, inflating the mean and SD without indicating superior etching uniformity; the same skew, to a lesser degree, is present across every other condition. This right-skew is shown directly in Figure 3, which plots every individual measurement per condition alongside the mean, median, and interquartile range.

#### 3.1.3. Elemental Composition (EDS)

Energy-dispersive X-ray spectroscopy (EDS) was used to probe the elemental composition of the etched products across the temperature series, with particular attention given to residual aluminium (a direct measure of the Al layer removal) and the fluorine and oxygen contents associated with surface terminations. Representative atomic percentages are summarised in Table 3.

All etched samples contain Ti, C, O, and F as their principal constituents, consistent with the formation of surface-terminated Ti_3_C_2_T_x_. The fluorine content increases markedly with etching temperature, from 12.2 at. % at 40 °C to 29.7 at. % at 80 °C, and then plateaus at 100 °C (29.3 at. %), indicating progressively more extensive fluorine content on the MXene surface as the thermal input increases [15]. The I(A_1g_)/I(E_g_) ratio reported in Section 3.3 does not show a monotonic trend with temperature, so this fluorine content trend is not independently corroborated by a corresponding monotonic change in Raman termination-band intensity.

Residual aluminium is highest at 40 °C (9.1 at. %), consistent with the incomplete, non-uniform etching identified at this temperature by SEM and XRD. At 60–100 °C, the residual Al content is substantially lower (2.1–4.7 at. %), confirming effective Al layer removal across this range. The Al signal does not, however, decrease monotonically with temperature: the lowest value is recorded at 60 °C. Minor amounts of chlorine (0.9–1.2 at. %) are detected in all samples, attributable to residual HCl-derived species from the etchant, consistent with the trace levels expected after the neutral pH washing protocol. A minor nitrogen signal (5.1 at.% at 80 °C, 0.8 at.% at 100 °C) was detected. Nitrogen incorporation during fluoride-salt-based in situ HF-generating etching has been reported in related MXene systems [35], though we note that this specific report concerns an acetic-acid-based route rather than NH_4_F/HCl directly, and the mechanism of nitrogen incorporation in our system remains to be confirmed.

The Al/Ti ratio, which normalises the aluminium content to the total titanium signal, provides an additional perspective on the dealumination extent. The precursor’s stoichiometric ratio (0.33) is essentially unchanged at 40 °C (0.35), confirming minimal Al layer removal at this temperature. Among the remaining conditions, however, Al/Ti alone does not track the overall assessment of etching quality developed in this study: 60 °C shows a lower Al/Ti ratio (0.06) than 80 °C (0.13), despite 60 °C showing a substantially lower fluorine content (17.0 vs. 29.7 at.% F) and a much higher residual-MAX XRD signature (composite index 3.74 vs. 1.81) than 80 °C. This reinforces a conclusion developed elsewhere in this study: dealumination alone, however it is normalised, is necessary but not sufficient to indicate high-quality MXene formation and must be considered alongside surface termination and structural order.

EDS was also performed across the etching duration series (30, 60, and 90 min, all at the fixed favourable temperature of 80 °C); the 30 min point corresponds to the 80 °C sample already reported above (Table 3). At 60 min, the aluminium content (3.7 at. %) is essentially unchanged from the 30 min value (3.6 at. %), while fluorine drops sharply to 7.4 at. %, less than a third of the 30 min value. At 90 min, aluminium falls further to 1.5 at. %, the lowest recorded across the duration series, while the fluorine content (11.4 at. %) remains well below the 30 min value, and oxygen returns at 7.7 at. %.

Although the aluminium content reaches its lowest value of the duration series at 90 min, this should not be read as indicating the most complete or highest-quality etching outcome: the fluorine content remains well below the 30 min value, and SEM (Section 3.1.2) shows increased heterogeneity at this duration, while XRD (Section 3.2.2) indicates that a substantial fraction of the analysed material remained unreacted, crystalline Ti_3_AlC_2_ rather than converted or degraded Ti_3_C_2_T_x_. Complete Al removal is therefore necessary but not sufficient for a well-formed MXene product; once dealumination is essentially complete, continued exposure does not straightforwardly improve the resulting structure.

Taken together, the duration series shows a marked decline in the fluorine content beyond 30 min (29.7 → 7.4 → 11.4 at. % at 30, 60, and 90 min, respectively) that is not mirrored by a corresponding recovery in aluminium removal, which remains low and non-monotonic across the same range (3.6, 3.7, and 1.5 at.% at 30, 60, and 90 min, respectively). This decoupling suggests that extended etching at 80 °C does not simply drive more complete surface fluorination and instead may promote loss or exchange of fluorine terminations with prolonged exposure [21]. As EDS reports bulk elemental atomic percentages rather than chemical bonding states, it cannot distinguish covalently bound Ti-F terminations from residual or adsorbed fluoride species; XPS analysis of the Ti-2p, F-1s, and O-1s core levels [36] would be needed to confirm whether this decline reflects true surface-termination loss rather than changes in residual fluoride retention during washing and is identified as a priority for future work on this system.

### 3.2. Structural Evolution and Phase Analysis (XRD)

X-ray diffraction was used to complement the morphological observations from SEM. Two reflections were used as primary diagnostic markers: the (002) basal reflection, whose position tracks interlayer spacing and shifts to lower 2θ upon successful delamination, and the (104) reflection, whose relative intensity tracks the amount of residual, unconverted Ti_3_AlC_2_ remaining in the sample. XRD of the unetched Ti_3_AlC_2_ precursor was collected and used as the primary reference for structural comparison. The measured diffractogram showed reflections at 2θ = 9.55°, 19.15°, 33.96°, 36.68°, 38.94°, 41.68°, 56.30°, and 60.05°, in close agreement with the reference pattern for hexagonal Ti_3_AlC_2_, ICDD Card No. 00-052-0875 [37], which reports characteristic reflections at 2θ ≈ 9.5° (002), 19.2° (004), 34.0° (101), 36.8° (103), 39.0° (104), 41.8° (105), 44.9° (106), 48.5° (107), 52.4° (108), 56.5° (109), and 60.3° (110). The precursor’s (002) reflection at 2θ = 9.55° corresponds to a basal spacing of 9.26 Å, consistent with the ICDD value of 9.30 Å.

#### 3.2.1. Effect of Etching Temperature on Phase Conversion

Relative to the unetched Ti_3_AlC_2_ precursor, whose measured (002) reflection lies at 2θ = 9.55° (d = 9.26 Å), consistent with ICDD 00-052-0875 [37], successful conversion to Ti_3_C_2_T_x_ is marked by a downward shift of the (002) reflection to 2θ ≈ 6.4–6.6° and the attenuation of the nonbasal (104) reflection at 2θ ≈ 39.0°, which reaches an intensity of 446 counts in the precursor. The corresponding expansion of the basal interlayer spacing from 9.26 Å to approximately 13.5–13.8 Å (a c-lattice parameter increase from 18.52 Å to ~27 Å) is consistent with the accommodation of surface terminations.

Every sample showed two coexisting features in the (002) region: a broad hump centred near 6.4–6.6°, consistent with the expanded basal spacing of converted Ti_3_C_2_T_x_, and a sharper peak at 9.4–9.5°, matching the unetched Ti_3_AlC_2_ (002) position. The coexistence indicates that etching to MXene occurs at every temperature investigated, but a substantial residual-MAX-phase fraction persists at lower temperatures. The sharp/broad ratio reported below quantifies the relative intensity balance between these two coexisting features—that is, the proportion of residual-MAX-phase to converted MXene signal—rather than the width or resolution of either peak individually; a lower ratio therefore indicates a smaller residual-MAX fraction, not a sharper or better-resolved MXene peak.

The balance between these two features shifted systematically with temperature (Table 4).

A sharp/broad ratio above 1 indicates that the unetched-MAX signature dominates the diffracting volume; below 1 indicates the converted MXene phase dominates. This places the crossover between MAX-dominant and MXene-dominant character between 60 °C and 80 °C, and the composite index—drawing on the full set of diagnostic reflections, unlike the (002) doublet alone—shows the same trend, falling by more than half between 60 °C and 80 °C before declining more modestly to 100 °C. Both metrics are therefore already close to their series minimum by 80 °C, indicating that the MAX-to-MXene phase transition is essentially complete at both 80 and 100 °C, with only a marginal further decrease at the higher temperature.

Alongside the SEM findings, this supports 80 °C as a generally better temperature within the range tested. The close similarity between 40 °C and 60 °C in both metrics is consistent with the SEM observation that morphological differences between these two conditions were not accompanied by a comparable degree of bulk crystallographic change.

The 80 °C sample also exhibits the largest basal expansion of the temperature series, with d(002) = 13.80 Å versus 13.50–13.55 Å at 40–60 °C, indicating the most fully developed interlayer spacing.

#### 3.2.2. Effect of Etching Time on Phase Conversion

The same coexistence behaviour was observed across the time series, conducted at the fixed temperature of 80 °C that we found to be most favourable. The 30 min time point corresponds to the 80 °C sample from Experiment 1 (Table 5).

At 60 min, the (002) region shows a broadened, intermediate feature bridging the two reference positions, and the sharp/broad ratio sits close to unity (1.09)—indicating a near-even mixture of converted and unconverted phase. The sample does not show a well-resolved MXene (002) reflection; instead, its low-angle region consists of a broad, weak hump centred near 2θ ≈ 8.7° (d ≈ 10.1 Å), only modestly expanded from the unetched-MAX spacing (9.30 Å) and reflecting poorly developed conversion at this intermediate duration relative to the much larger ~13.8 Å spacing seen at 30 min. This poorly developed basal reflection, together with the near-unity sharp/broad ratio (1.09), indicates an incompletely converted sample at this duration.

At 90 min, both metrics reverse sharply: the sharp/broad ratio rises to 1.79, and the composite index reaches 5.75—the highest value recorded across the entire dataset, exceeding even the least-etched 40 °C sample. This is not consistent with disordered or degraded Ti_3_C_2_T_x_: the (104) region signal at 90 min resolves as a sharp doublet at 2θ ≈ 38.68° and 38.88°, closely matching the splitting of the doublet observed in the pristine Ti_3_AlC_2_ precursor (2θ ≈ 38.74° and 38.94°), offset by only ~0.06° toward a lower angle. Using the same intensity-to-background convention applied throughout this study (Section 2.5), the 90 min (104) signal reaches an I/background ratio approximately 1.2 times that of the precursor’s own (104) reflection, indicating an intensity at least comparable to, and somewhat exceeding, the crystalline precursor rather than a weakened or broadened feature. Disordered or defect-rich material would be expected to produce broadened, weakened diffraction features rather than a sharp, well-resolved doublet at least matching the crystalline precursor’s own intensity. We therefore interpret the elevated composite index at 90 min as evidence of a substantial population of genuinely unreacted, crystalline Ti_3_AlC_2_ remaining in the analysed material, consistent with the dense, poorly layered particles identified by SEM at this duration (Section 3.1.2) being unetched-MAX-phase cores rather than degraded MXene. This interpretation is compatible with the EDS-measured aluminium content at 90 min (Section 3.1.3): energy-dispersive X-ray spectroscopy in SEM samples a shallow near-surface interaction volume (on the order of 1–2 µm), whereas laboratory XRD integrates over a substantially greater bulk depth. A particle population with a well-etched, Al-depleted surface overlying an unreacted MAX-phase core would therefore be expected to show low surface Al by EDS while still contributing strongly to bulk MAX-phase diffraction signals, without requiring any reversal or degradation of previously converted material. The XRD and SEM data are therefore in agreement that 90 min is the least favourable condition in the time series, with a substantial unreacted-core population rather than uniform conversion [21].

It is worth clarifying that the (104) reflection and the other diagnostic reflections contributing to the composite index report on the presence of crystallographically ordered Ti_3_AlC_2_ (MAX-phase) structure, whereas EDS reports the elemental composition of a shallow, near-surface sampled volume. The elevated composite index at 90 min, despite the lowest EDS-measured aluminium content of the duration series (1.5 at.%), is therefore consistent with a population of particles bearing an etched, Al-depleted surface region over a substantially unreacted, aluminium-rich Ti_3_AlC_2_ core, rather than with disordered or degraded Ti_3_C_2_T_x_: as noted above, the sharp doublet structure and intensity of the 90 min (104) signal closely resemble crystalline Ti_3_AlC_2_ rather than a disordered diffracting phase [38].

#### 3.2.3. Cross-Correlation with SEM and Identification of Favourable Conditions

**Figure 4:**
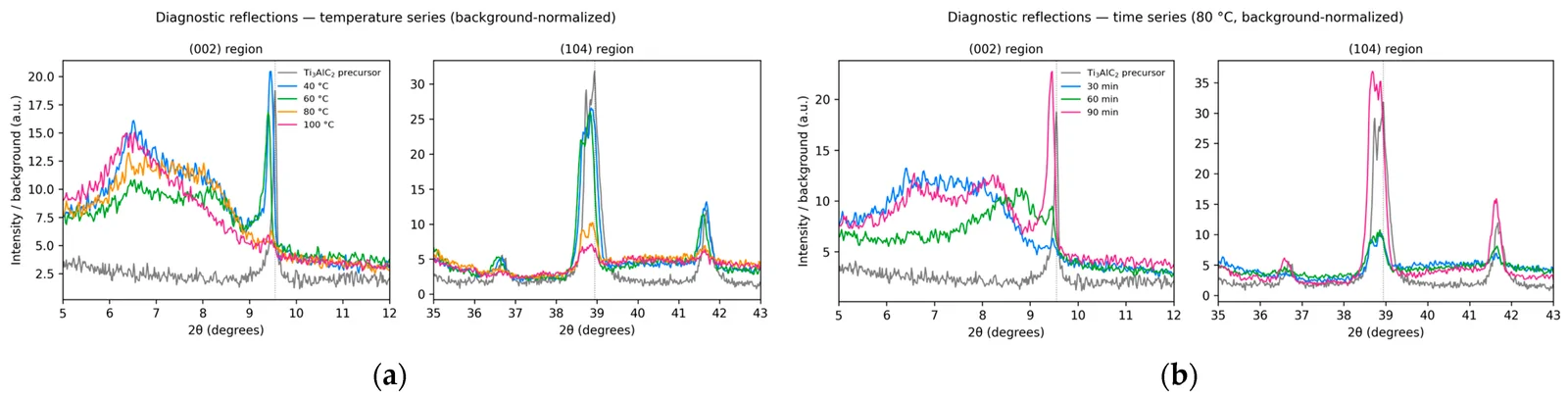
XRD diagnostic reflections with unetched Ti_3_AlC_2_ precursor (grey) shown for direct comparison. (**a**) Temperature series, (**b**) time series. The sharp/broad ratio and composite residual-MAX index (Table 4 and Table 5), calculated using the same intensity-to-background convention, remain the primary quantitative metrics for cross-condition comparison. Thin vertical lines mark the (002) and (104) reflection positions of the Ti_3_AlC_2_ precursor.

Within the parameter space investigated, the XRD and SEM datasets converge on 80 °C, 30 min as the most favourable region, with the composite residual-MAX index and the (002) sharp/broad ratio both identifying this region as the point where the converted MXene phase becomes dominant over residual unetched material without the structural degradation observed by SEM at 100 °C (Figure 4).

Considered in isolation, the sharp/broad ratio and composite residual-MAX index are marginally lower at 100 °C (0.39, 1.62) than at 80 °C (0.47, 1.81), indicating a slightly smaller residual-MAX diffracting fraction at the higher temperature. This should not be read as evidence that 100 °C represents a structurally superior condition. Both values are close to their series minimum by 80 °C, indicating that the MAX-to-MXene phase transition is essentially complete at both temperatures, and the further decrease at 100 °C is small relative to the more than twofold improvement already achieved between 60 and 80 °C. By a more direct structural measure, the 80 °C sample instead shows the largest basal-plane expansion of the entire series (d(002) = 13.80 Å, versus 13.50 Å at 100 °C, which reverts to the value seen at the less-converted 60 °C condition), indicating the most fully developed interlayer spacing at 80 °C rather than 100 °C. Combined with the fragmentation and edge erosion observed by SEM at 100 °C—a form of degradation that XRD phase-fraction metrics do not directly capture—and the absence of any compositional advantage in EDS (the F and Al contents are similar between 80 and 100 °C), the cross-technique evidence indicates that 80 °C, not 100 °C, represents the more favourable condition despite the XRD phase-fraction metrics showing a marginal further decrease at the higher temperature.

### 3.3. Structural and Defect Analysis (Raman Spectroscopy)

**Figure 5:**
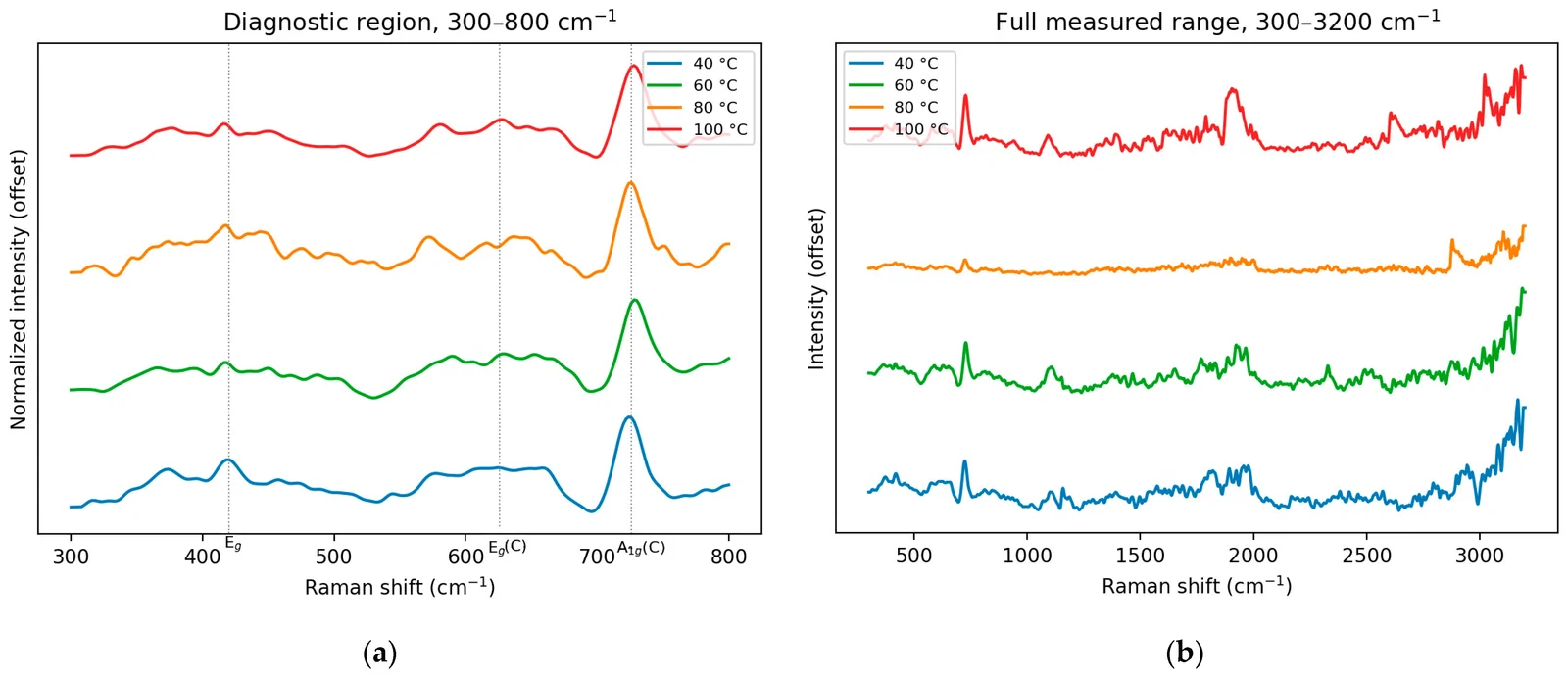
Raman spectra of Ti_3_C_2_T_x_ synthesised at 40, 60, 80, and 100 °C (30 min etching). (**a**) Resolved diagnostic region (300–800 cm^−1^), baseline-subtracted, normalised to the A_1g_ (C) peak height (**b**) full measured range (300–3200 cm^−1^). Dotted vertical lines mark the guide for the E_g_, E_g_(C), and A_1g_(C) band positions.

Raman spectroscopy was used to probe the vibrational structure and surface terminations of the etched samples across the full temperature series (40, 60, 80, and 100 °C; 30 min etching). Spectra were acquired over 300–3200 cm^−1^; the diagnostic Ti_3_C_2_T_x_ modes lie below 800 cm^−1^ and are shown in the resolved region (Figure 5a), with the full measured range (Figure 5b). Peak heights were measured relative to a linear baseline anchored at 300 and 800 cm^−1^, a region common to all four spectra that brackets the diagnostic band envelope without intersecting any resolved feature.

All four samples exhibit the characteristic vibrational fingerprint of Ti_3_C_2_T_x_. Three principal features are resolved: a broad E_g_ band near 416—420 cm^−1^, assigned to in-plane vibrations of surface-termination groups (-O, -OH, -F) bonded to the outer Ti atoms; an E_g_ (C) band at 624–636 cm^−1^; and a prominent A_1g_ (C) mode at 724—728 cm^−1^ [23,28]. The presence of the A_1g_ (C) mode in all four samples confirms the formation of the Ti_3_C_2_ core structure across the full temperature range investigated. Diagnostic band positions are essentially invariant with temperature (Eg: 416–420 cm^−1^; A_1g_ (C): 724–728 cm^−1^), indicating that the core Ti_3_C_2_ bonding environment is preserved across the series. The ratio of the A_1g_ (C) mode to the termination-associated E_g_ band, I(A A_1g_)/I(E_g_), is 1.90 at 40 °C, rises to a maximum of 3.28 at 60 °C, returns to 1.90 at 80 °C, and reaches 2.84 at 100 °C.

The full width at half maximum of the A_1g_ (C) mode was 22–24 cm^−1^ across all four conditions, showing no resolvable broadening or narrowing with temperature. Above ~1000 cm^−1^, all spectra show a rising, weakly structured background extending to the upper measurement limit, attributed to photoluminescent/fluorescence background rather than to discrete vibrational modes [34]. No sharp, well-defined bands attributable to crystalline TiO_2_ were resolved within the measured window; however, the anatase diagnostic region below 300 cm^−1^ fell outside the acquisition range [39,40].

**Figure 6:**
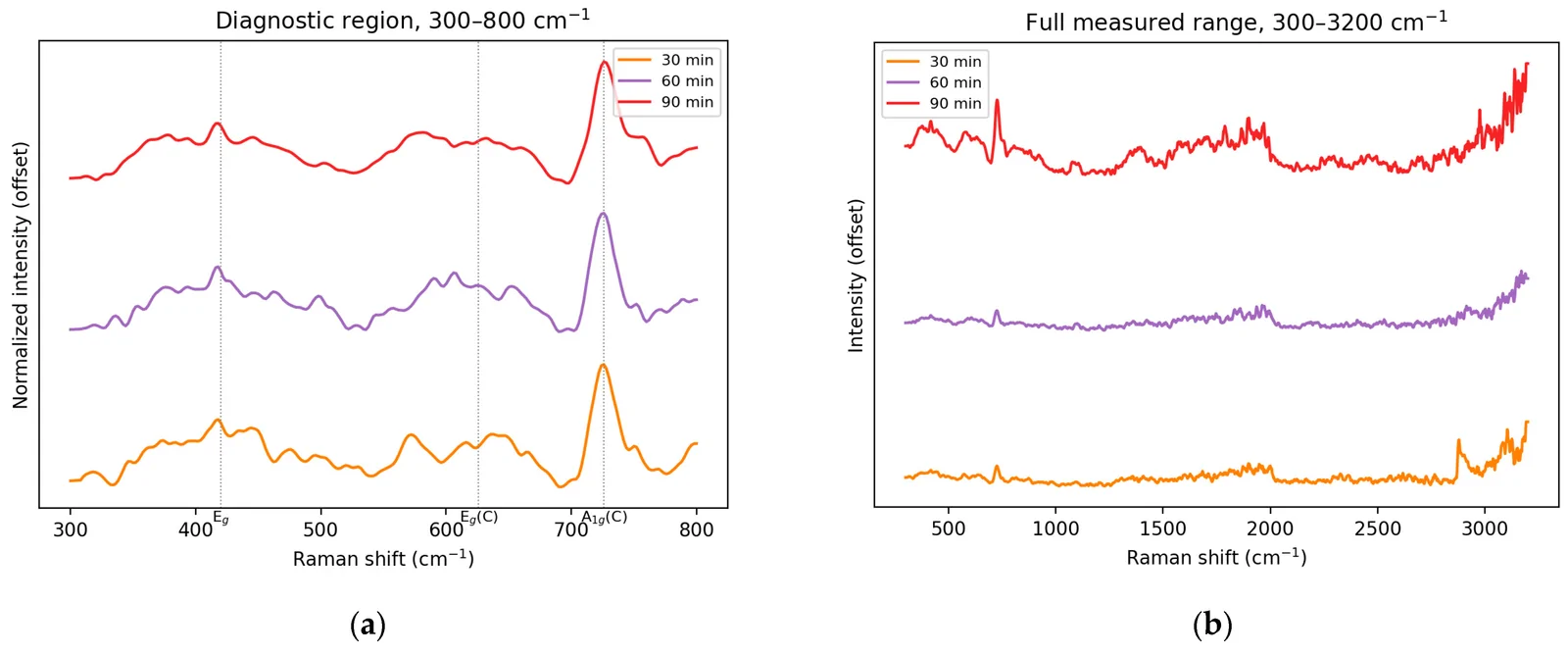
Raman spectra of Ti_3_C_2_T_x_ synthesised at 80 °C for 30, 60, and 90 min. (**a**) Resolved diagnostic region (300–800 cm^−1^); (**b**) full measured range (300–3200 cm^−1^). Dotted vertical lines mark the guide for the E_g_, E_g_(C), and A_1g_(C) band positions.

Raman spectroscopy was also performed across the etching duration series (Figure 6), and all three durations retain the full Ti_3_C_2_T_x_ diagnostic fingerprint, with the E_g_, E_g_ (C), and A_1g_ (C) modes resolved at essentially unchanged positions (Eg: 418 cm^−1^; A_1g_ (C): 726 cm^−1^) across all three durations, indicating that the core carbide bonding environment is not measurably altered by the etching time in this range. The I(A_1g_)/I(E_g_) ratio is 1.90 at 30 min, changes only marginally to 1.85 at 60 min, and rises to 2.12 at 90 min [41].

The 90 min sample does show a modestly higher I(A_1g_)/I(E_g_) ratio and a slightly broader A_1g_ (C) mode (FWHM 24 cm^−1^, versus 22 cm^−1^ at 30 and 60 min) than the shorter durations, which is at least directionally consistent with the XRD- and SEM-based identification of 90 min as the least favourable condition in the duration series.

## 4. Discussion

The non-monotonic trends observed in both the temperature and duration series point to at least two competing processes governing the etching outcome, rather than a single rate-limiting step. In the temperature series, residual aluminium reached its lowest value at 60 °C (2.1 at. %) rather than declining monotonically toward 100 °C, while the fluorine content continued rising through 80 °C before plateauing. This decoupling suggests that Al layer dissolution and surface fluorination do not share the same temperature dependence: Al removal may approach completion by 60 °C, while fluorine termination coverage continues to build with additional thermal input up to 80 °C. A similar decoupling was observed across the duration series, where the fluorine content fell sharply beyond 30 min without a corresponding gain in aluminium removal. Taken together, these results suggest that once Al removal is largely complete, continued exposure to the NH_4_F/HCl etchant does not drive further conversion but instead promotes the loss or exchange of existing surface terminations. The SEM- and XRD-based observations at 90 min are consistent with this interpretation only in part: increasing heterogeneity and a growing population of dense, poorly layered particles are observed by SEM, but the XRD signature at this duration (Section 3.2.2) instead indicates a substantial unreacted, crystalline Ti_3_AlC_2_ fraction rather than degradation of already-formed MXene sheets. We note that a longer etching time cannot, on its own, be expected to leave more unreacted MAX phase than a shorter one; the most likely explanation is that the single aliquot characterised at 90 min was not fully representative of the pooled batch material at that condition, rather than a genuine reversal of conversion with duration. Because individual batches were not characterised separately (Section 2.4), this aliquot-level heterogeneity cannot be distinguished from a true duration effect in the present dataset, and we identify separately characterised replicate batches as the necessary next step to resolve this. This is distinct from the degradation mechanism identified at 100 °C (below), where etching is essentially complete and continued thermal input instead damages already-converted MXene, consistent with reports that prolonged or aggressive etching conditions can degrade and disorder previously converted Ti_3_C_2_T_x_ [21,42]. Nor do we interpret the elevated 90 min composite index as evidence of true MAX-phase recrystallisation, which would be thermodynamically atypical under these mild aqueous conditions and is not otherwise documented for NH_4_F/HCl-type etching systems. Raman spectroscopy did not resolve these effects as clearly as SEM, XRD, and EDS. This study represents a screening investigation across four temperatures and three durations. The parameter grid is relatively coarse, and the true optimum may lie between the sampled points rather than exactly at 80 °C/30 min. The reproducibility of the 80 °C/30 min condition across independent synthesis batches was not directly assessed via separately characterised replicates. Material from four independent batches was pooled prior to characterisation throughout this study (Section 2.4) specifically to reduce the effect of local sample heterogeneity on the reported values; this pooling approach does not itself demonstrate or establish batch-to-batch reproducibility, since individual batches were not characterised separately. Independent characterisation of replicate batches is identified as a necessary next step. Surface-termination chemistry was inferred from EDS elemental composition; as XPS access was not available for this study, the fluorine and oxygen trends reported here should be interpreted as compositional indicators of termination-related change rather than direct evidence of specific Ti–F or Ti–O bonding states, and XPS confirmation is an important next step to quantify F-loss inferred from EDS [43].

The deterioration observed at higher etching temperatures should not be interpreted as incomplete or insufficient etching. By 100 °C, Al removal is already essentially complete (4.7 at.% residual Al, similar to 60–80 °C and far below the 9.1 at.% seen at 40 °C), and both the XRD composite residual-MAX index (1.62) and the (002) sharp/broad ratio (0.39) reach their lowest values of the temperature series, indicating the least residual-MAX-phase signature of any condition tested, consistent with the (104)-based residual-MAX tracking reported for other in situ HF-generating systems such as LiF + HCl [44]. The fluorine content also remains near its series maximum (29.3 at.%, similar to 80 °C), showing that surface termination is not reduced at 100 °C. Rather than reflecting incomplete conversion, the fragmentation, edge erosion, and increased surface debris observed by SEM at 100 °C are consistent with over-etching: once the Al layer dissolution is largely complete, continued thermal input no longer drives further MAX-to-MXene conversion but instead accelerates the degradation of the already-formed Ti_3_C_2_T_x_ sheets, promoting edge damage and structural breakdown of the thin, newly exposed lamellae. This is consistent with reports that aggressive or excessive etching conditions—whether via elevated HF concentration, extended duration, or here, elevated temperature—degrade rather than improve MXene structural quality once the underlying MAX phase has been consumed [21,45].

Raman characterisation in this study was constrained by the instrument range: the spectrometer used (Bruker Bravo, Duo Laser™ excitation) has a manufacturer-specified spectral range of 300–3200 cm^−1^ and cannot access the ~200 cm^−1^ region reported by Sarycheva and Gogotsi [34] and Parker et al. [46] as the most intense Ti_3_C_2_T_x_ Raman feature. The E_g_ and A_1g_(C) modes analysed here (416–420 and 724–728 cm^−1^) remain independently established diagnostic markers of the Ti_3_C_2_T_x_ structure within the accessible range [34], and their consistent presence across all seven synthesis conditions supports the structural conclusions drawn from SEM, XRD, and EDS. Extending the Raman characterisation below 300 cm^−1^ to capture the full diagnostic fingerprint, including the ~200 cm^−1^ mode, would require a different instrument and is identified as a priority for future work on this system.

Finally, this study characterises structural and compositional outcomes, rather than direct applications.

## 5. Conclusions

This study systematically varied the etching temperature (40–100 °C) and etching duration (30–90 min) for the NH_4_F/HCl, microwave-assisted synthesis of Ti_3_C_2_T_x_ MXene from Ti_3_AlC_2_, using SEM, EDS, XRD, and Raman spectroscopy to identify a favourable processing window.

Across the temperature series, SEM showed accordion-like layering becoming progressively more uniform from 40 to 80 °C and then degrading at 100 °C. XRD showed the composite residual-MAX index and the sharp/broad ratio both placing the crossover from MAX-dominant to MXene-dominant character between 60 and 80 °C, with the 80 °C sample showing the largest basal expansion of the series. EDS showed residual aluminium dropping sharply beyond 40 °C, with a non-monotonic minimum at 60 °C. The duration series identified 80 °C, 30 min as the most favourable condition: extending etching to 60 and 90 min did not improve outcomes, with SEM showing increasing heterogeneity and a growing population of dense layered particles and XRD showing the sharp/broad ratio and composite residual-MAX index both rising again at 90 min, reaching values higher than any point in the temperature series, including the least-etched 40 °C sample. EDS showed the fluorine content falling sharply beyond 30 min without a corresponding gain in aluminium removal. SEM, XRD, and EDS independently identified 90 min as the least favourable duration tested. Raman spectroscopy showed that the band positions are invariant across both series, indicating that the core Ti_3_C_2_ bonding environment forms under all conditions tested, even where SEM and XRD identify a sample as poorly converted overall. Taken together, the three techniques agree that 80 °C and 30 min represent the most favourable conditions within the parameter space investigated for NH_4_F/HCl, microwave-assisted etching of Ti_3_AlC_2_ and that 90 min was the least favourable duration tested by every metric that responded to it. As discussed above, this should not be read as establishing that extending the etching time itself actively degrades the product: the XRD signature at 90 min is more consistent with a substantial unreacted-core fraction than with the degradation of converted material, and given that individual synthesis batches were not characterised separately, this result cannot be fully distinguished from aliquot-level heterogeneity at this single duration. These findings should be interpreted as a screening study across four temperatures and three durations. Confirming the 80 °C/30 min window with replicate batches and extending the matrix to finer temperature and time intervals around this point would be a useful next step toward a reproducible, safer, and time-efficient NH_4_F-based protocol for Ti_3_C_2_T_x_ production.

## Figures and Tables

**Table 1:** Experimental design for the two etching series.

Parameter	Experiment 1 (Temperature Series)	Experiment 2 (Duration Series)
Etching temperature	40, 60, 80, 100 °C (varied)	80 °C
Etching duration	30 min (fixed)	30, 60, 90 min (varied)
Ramp time	25 min	25 min
Power	300 W	300 W
Purpose	Identify favourable etching temperature	Evaluate effect of etching time at favourable temperature

**Table 2:** SEM-derived interlayer spacing measurements across the etching temperature series and the etching duration series.

Condition	n	Median (nm)	IQR (nm)	Mean ± SD (nm)	Range (nm)
40 °C	13	310	212–457	385 ± 279	98–1176
60 °C	14	670	404–1456	967 ± 659	299–2244
80 °C (=30 min)	12	516	373–888	668 ± 383	295–1604
100 °C	14	524	316–848	622 ± 400	131–1457
60 min	13	395	368–423	416 ± 87	314–661
90 min	12	290	253–423	337 ± 167	136–742

**Table 3:** EDS elemental composition (at. %) of etched Ti_3_C_2_T_x_ across the temperature series (40–100 °C, 30 min) and the etching duration series with comparison to the precursor MAX phase. The 80 °C/30 min column is shared between both series.

Element (at. %)	MAX Phase Ti_3_AlC_2_	40 °C	60 °C	80 °C (=30 min)	100 °C	60 min	90 min
Ti	50.0	26.2	36.7	28.2	26.8	80.4	69.5
C	33.3	16.5	27.8	13.9	19.0	7.8	8.1
O	-	35.1	15.3	18.3	18.4	0.2	7.7
F	-	12.2	17.0	29.7	29.3	7.4	11.4
Al	16.7	9.1	2.1	3.6	4.7	3.7	1.5
Cl	-	0.9	1.1	1.2	1.0	0.5	1.8
N	-	-	-	5.1	0.8	-	-

**Table 4:** XRD-derived (002) peak position, interlayer spacing, sharp/broad ratio, and composite residual-MAX index across the etching temperature series (30 min etching).

Temperature (°C)	(002) 2θ (°)	d(002) (Å)	(002) Sharp/Broad Ratio	Composite Residual-MAX Index
40	6.52	13.55	1.27	4.47
60	6.54	13.50	1.55	3.74
80	6.40	13.80	0.47	1.81
100	6.54	13.50	0.39	1.62

**Table 5:** XRD-derived (002) peak position, interlayer spacing, sharp/broad ratio, and composite residual-MAX index across the etching duration series (80 °C).

Duration (min)	(002) 2θ (°)	d(002) (Å)	(002) Sharp/Broad Ratio	Composite Residual-MAX Index
30 (=80 °C)	6.40	13.80	0.47	1.81
60	~8.7 (broad)	~10.1	1.09	2.00
90	6.58	13.42	1.79	5.75

## Data Availability

The original contributions presented in the study are included in the article/Supplementary Materials. Further inquiries can be directed to the corresponding authors.

## Supplementary Materials

The following supporting information can be downloaded at: https://www.mdpi.com/article/10.3390/ma19194172/s1, Figure S1: Low-magnification SEM survey images of the etching-temperature series (40–100 °C): (a) 40 °C; (b) 60 °C; (c) 80 °C; (d) 100 °C. All images were acquired at 2500× magnification (horizontal field width 166 μm) in secondary-electron (SE) mode.

## Abbreviations

The following abbreviations are used in this manuscript:

XPS	X-ray photoelectron spectroscopy
DI	deionised
IQR	interquartile range
EDS	energy-dispersive X-ray spectroscopy
EMI	electromagnetic interference
FWHM	full width at half maximum
HCl	hydrochloric acid
HF	hydrofluoric acid
ICDD	International Centre for Diffraction Data
MAX	M_n+1_AX_n_ phase (early transition metal, group A element, carbon/nitrogen)
PPE	personal protective equipment
SD	standard deviation
SEM	scanning electron microscopy
XRD	X-ray diffraction
