# Supplementary material for: Effect of Etching Temperature and Time on In Situ HF-Generating (NH4F) Synthesis of Ti3C2Tx MXene
Source: Materials (Basel). 2026 Sep 29;19(19):4172. doi: 10.3390/ma19194172 (PMC13665931; doi:10.3390/ma19194172)
Supplement: Supplementary file 1 [file materials-19-04172-s001.zip › materials-4543542-supplementary.pdf]

Supplementary Materials

Paper title: Effect of Etching Temperature and Time on In Situ HF-Generating (NH_4F) Synthesis of Ti_3C_2Tx MXene

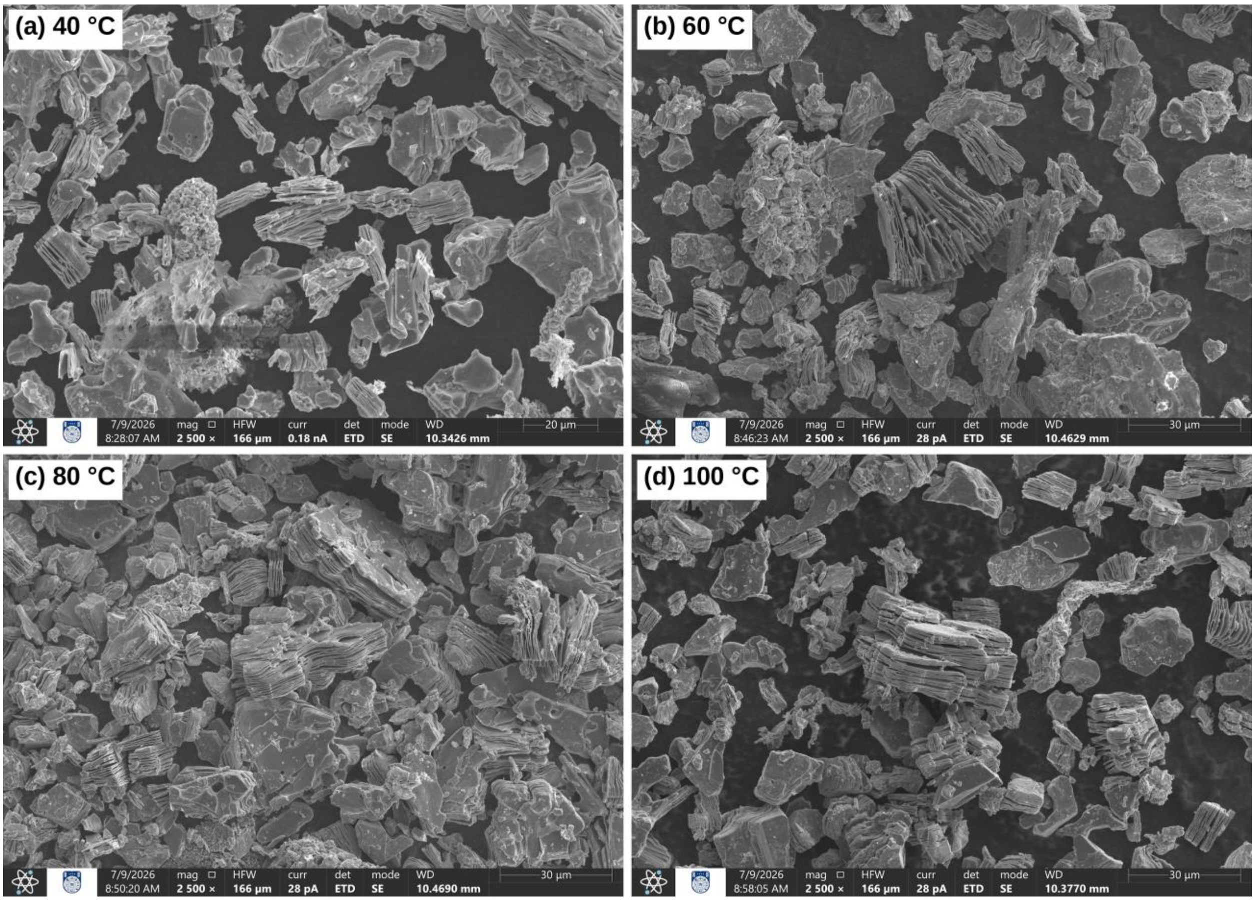

Figure S1. Low-magnification SEM survey images of the etching-temperature series (40–100 °C): (a) 40 °C; (b) 60 °C; (c) 80 °C; (d) 100 °C. All images were acquired at 2500× magnification (horizontal field width 166 μm) in secondary-electron (SE) mode.
